# An application of propensity score weighting to quantify the causal effect of rectal sexually transmitted infections on incident HIV among men who have sex with men

**DOI:** 10.1186/s12874-015-0017-y

**Published:** 2015-03-21

**Authors:** Adam S Vaughan, Colleen F Kelley, Nicole Luisi, Carlos del Rio, Patrick S Sullivan, Eli S Rosenberg

**Affiliations:** Department of Epidemiology, Rollins School of Public Health, Emory University, 1518 Clifton Rd NE, Atlanta, 30322 GA USA; Division of Infectious Diseases, Department of Medicine, Emory University School of Medicine, Atlanta, GA USA; Hubert Department of Global Health, Rollins School of Public Health, Emory University, Atlanta, GA USA

**Keywords:** STI, HIV, Propensity scores, Survival analysis, Men who have sex with men, Marginal structural models

## Abstract

**Background:**

Exploring causal associations in HIV research requires careful consideration of numerous epidemiologic limitations. First, a primary cause of HIV, unprotected anal intercourse (UAI), is time-varying and, if it is also associated with an exposure of interest, may be on a confounding path. Second, HIV is a rare outcome, even in high-risk populations. Finally, for most causal, non-preventive exposures, a randomized trial is impossible. In order to address these limitations and provide a practical illustration of efficient statistical control via propensity-score weighting, we examine the causal association between rectal STI and HIV acquisition in the InvolveMENt study, a cohort of Atlanta-area men who have sex with men (MSM). We hypothesized that, after controlling for potentially confounding behavioral and demographic factors, the significant STI-HIV association would attenuate, but yield an estimate of the causal effect.

**Methods:**

The exposure of interest was incident rectal gonorrhea or chlamydia infection; the outcome was incident HIV infection. To adjust for behavioral confounding, while accounting for limited HIV infections, we used an inverse probability of treatment weighted (IPTW) Cox proportional hazards (PH) model for incident HIV. Weights were derived from propensity score modeling of the probability of incident rectal STI as a function of potential confounders, including UAI in the interval of rectal STI acquisition/censoring.

**Results:**

Of 556 HIV-negative MSM at baseline, 552 (99%) men were included in this analysis. 79 men were diagnosed with an incident rectal STI and 26 with HIV. 6 HIV-infected men were previously diagnosed with a rectal STI. In unadjusted analysis, incident rectal STI was significantly associated with subsequent incident HIV (HR (95%CI): 3.6 (1.4-9.2)). In the final weighted and adjusted model, the association was attenuated and more precise (HR (95% CI): 2.7 (1.2-6.4)).

**Conclusions:**

We found that, controlling for time-varying risk behaviors and time-invariant demographic factors, diagnosis with HIV was significantly associated with prior diagnosis of rectal CT or GC. Our analysis lends support to the causal effect of incident rectal STI on HIV diagnosis and provides a framework for similar analyses of HIV incidence.

**Electronic supplementary material:**

The online version of this article (doi:10.1186/s12874-015-0017-y) contains supplementary material, which is available to authorized users.

## Background

As with many preventive exposures, causal associations between preventive exposures (e.g. pharmaceuticals) and incident HIV may be optimally assessed through randomized clinical trials (RCT). However, causal associations between non-preventive exposures, such as high-risk sexual or substance use behaviors, and incident HIV cannot be ethically evaluated using an RCT. Additional limitations may further increase the analytic complexities of assessing these causal relationships. First, high-risk sex behaviors, including unprotected anal intercourse (UAI), receptive anal intercourse (RAI), and partners selected from a high HIV prevalence pool, are time-varying and, as necessary causes, must occur in an interval prior to HIV diagnosis [1]. Given an HIV-related exposure of interest that similarly requires high-risk sex risk behaviors (such as anal trauma or another sexually transmitted infection), these behaviors must be modeled as time-varying factors that may be on a confounding path [2]. Additionally, regression-based incidence analyses are further challenged by limited statistical power due to relatively small numbers of incident HIV, even among high-risk populations. Recent studies have found annual HIV incidence of MSM in urban areas of the United States of 1-7%, requiring large cohorts observed for long periods of time to accumulate sufficient events for analysis [3-7].

Consequently, in the absence of RCT data, establishing a causal association between a non-preventive exposure and incident HIV requires application of epidemiologic analysis methods that can control for time-varying behavioral confounding and accommodate small numbers of observed events. The ideal data would include longitudinal evaluation of the exposure, outcome, and all possible time-varying confounders, with measurements made using methods to minimize misclassification. Given such a dataset, propensity score methods would address these issues and, with confirmation of model assumptions, provide an estimate of the causal effect of interest [8].

**Figure 1:**
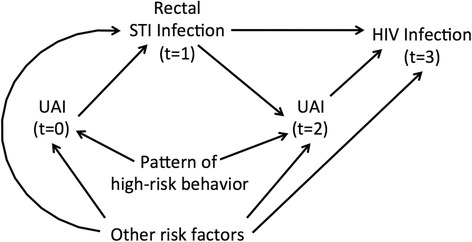
**Directed acyclic graph (DAG) illustrating the hypothesized rectal STI-HIV association and time-varying behavioral confounding.**

An important example of these analytic limitations is the potential causal association between HIV and sexually transmitted infections (STI). Although recent studies have observed associations between these two infections, evaluating a casual association requires adequate control of high-risk sex in the time period immediately preceding both STI and HIV diagnosis and of patterns of high-risk behaviors [3-7,9,10]. Additionally, sexual history in the interval prior to each diagnosis is required to best control for the potentially time-varying nature of the confounding and to account for changes in behavior that may result from rectal STI diagnosis (Figure 1) [11-13]. Therefore, to address these analytic requirements, we detail the application of propensity score weighting to examine the causal effect of rectal bacterial STI on HIV acquisition [8]. We use a cohort of Atlanta-area MSM with biologically measured exposure (i.e. incident STI) and outcome (i.e. incident HIV) data and longitudinal measures of confounders (i.e. high-risk sex). This report serves as an applied methodological exposition of propensity score weighting that pairs with a forthcoming clinically-oriented report on the associations between a broader set of STIs (including urethral bacterial STIs and syphilis) and HIV [14]. We hypothesized that, controlling for time-varying and time-invariant behavioral and demographic factors, the positive association between rectal STI and subsequent HIV infection would be mitigated, yielding an estimate of the causal effect.

## Methods

### Data sources and definitions

The InvolveMENt study is a recently concluded, longitudinal cohort of black and white, sexually active MSM aged 18–39 years in Atlanta, Georgia, recruited from community-based venues and social media between June 2010 through October 2012 [15]. MSM were tested for HIV at enrollment using rapid antibody tests, with confirmatory serum CD4 and viral load measures for preliminary positives. MSM who were HIV-negative at enrollment were included in the longitudinal portion of the study, attending study visits at 3 and 6 months, and subsequent 6 month intervals for two years, or until HIV seroconversion. At the same visits, participants were tested for urethral and rectal *Neisseria gonorrhoeae* (gonorrhea) and *Chlamydia trachomatis* (chlamydia) using nucleic acid amplification testing and *Treponema pallidum* (syphilis). using the rapid plasma regain (RPR) test with confirmatory quantitative nontreponemal titers and treponemal IgG [16,17]. At each study visit, participants completed a computer-administered questionnaire that collected aggregate sexual behaviors, such as the number of UAI partners, and included a dyadic inventory of the most recent 5 sex partners in the previous 6 months [15]. Demographic and sexual behaviors (i.e.: condom use, receptive and insertive sex roles) were collected for each of these partners.

For this analysis, the outcome was incident HIV infection. The exposure was defined as the first (i.e. earliest) diagnosis of incident rectal STI (either gonorrhea or chlamydia). An STI diagnosis was considered to be incident if the individual tested negative for the same STI in the prior interval, or if the STI diagnosis followed an initial visit with the same STI diagnosis with confirmation of study-provided treatment. As we could not determine the timing of the rectal STI for men who were diagnosed with a rectal STI at the initial study visit, we did not include these infections in the analysis. For individuals with incident STI, person-time was calculated as the difference between the date of STI diagnosis and the date of HIV seroconversion or censoring; for individuals without an STI, person-time was calculated as the difference between the enrollment date and the date of HIV seroconversion or censoring due to study completion or loss to follow-up. The date of HIV seroconversion was estimated as halfway between the dates of the final (ie: seroconversion) visit and penultimate visits [18].

### Analysis methods

A crude hazard ratio (HR) for the association between incident rectal STI and incident HIV was calculated using an unadjusted Cox proportional hazards (PH) model.

To adjust for behavioral confounding of the rectal STI-HIV association, while accounting for a limited number of incident HIV infections, we used an inverse probability of treatment weighted (IPTW) Cox proportional hazards (PH) model for incident HIV, where the weights were derived from propensity score modeling of STI incidence (i.e. a marginal structural model) [19]. We note that the propensity score literature typically employs the word ‘treatment’ to differentiate the two exposure groups. As our exposure is not a treatment, we use the term ‘exposure groups’ rather than ‘treatment groups’. We first conceptually outline the approach as a four-step process and then detail the specific application to the rectal STI-HIV association.

### Inverse probability of treatment weighted (IPTW) Cox proportional hazards (PH) modeling

#### Propensity score estimation

When applied to observational data, properly specified propensity scores simulate the gold-standard of epidemiologic studies, the RCT [8]. In an RCT, all potential confounders, both measured and unmeasured, are, on average, evenly distributed across (and thus independent of) exposure status.

However, using non-randomized observational data, as in our analysis, exposure status may be associated with measured and unmeasured covariates. Propensity score estimation begins by modeling exposure status (generally using logistic regression) as a function of potential confounders of the exposure and outcome. In the case of behavioral confounding, these potential confounders should temporally precede both the exposure and the outcome and be associated with both. This model then estimates probabilities of exposure conditional on a set of measured preceding covariates, or “propensity scores”. These scores may be incorporated in subsequent covariate-adjusted, stratified, matched, or weighted analyses in order to balance covariates across exposure groups in an observational study or to control residual confounding resulting from a failure of randomization [8,20].

In time-to-event analyses, applying propensity scores using inverse probability of treatment weights (IPTW) minimizes bias relative to the other methods of applying propensity scores [21]. IPTW are defined as the inverse of the propensity score. In order to reduce the influence of outlying weights (i.e. those observations with a very high or very low propensity score), weights may be stabilized via multiplication by the mean propensity score of the given exposure group [19,22].

#### Common support assessment

The degree to which the propensity score has been appropriately specified may initially be ascertained through evaluation of common support. Common support is defined by overlapping distributions of propensity scores between exposure groups. Unlike an RCT, confounding in an observational study will almost certainly lead to different distributions of propensity scores between exposure groups. However, overlap in the distributions indicates the potential for a member of the exposed group to be in the unexposed group and that individuals with each level of covariates may have either exposure status (i.e. supporting the assumptions of exchangeability and positivity) [23]. A lack of common support, or a complete separation of propensity scores between the exposure groups indicates severe differences between the two exposure groups and the possibility that confounding cannot be reduced using propensity methods [22].

#### Balance assessment

Given common support, the degree to which confounding by the modeled factors has been controlled may be assessed by examining balance, or distribution of potential confounders by exposure status. Balance is assessed after applying propensity scores to the sample in a method analogous to that use in the final exposure effect estimate (i.e. apply IPTW to the sample before assess balance). Balance is most often examined using standardized bias, calculated as the difference in mean covariate value between exposure groups, divided by the standard deviation of the covariate in the entire study sample following application of propensity scores [8]. While a standardized bias <0.25 is considered to indicate balance of the potential confounder between exposure groups, others have proposed a threshold of <0.10 [24,25]. In the absence of balanced potential confounders following application of propensity scores, the final effect estimate is prone to residual confounding and, therefore, a better propensity model should be developed. Statistical testing is inappropriate for assessing balance between treatment groups because balance is a property of the sample and not of an underlying population [26,27].

#### Weighted survival analysis

Application of propensity scores from a model exhibiting both common support and balance will reduce or eliminate confounding by those measured covariates. For time-to-event analyses, application of propensity scores using IPTW (rather than matching, stratification, or adjustment) produces effect estimates with minimal bias [21]. When applied to a Cox PH model, IPTW creates a pseudo-population that permits estimation of the casual effect of the exposure on the outcome, given that all confounders were appropriately accounted for in the propensity model. These models may also be further adjusted by predictors of the outcome to increase precision of the final effect estimate.

### Application: estimating the rectal STI-HIV association

#### Propensity score estimation

Using logistic regression, we modeled the probability of exposure (incident rectal STI) as a function of the following potential confounders: participant’s race, participant’s age at rectal STI diagnosis/censoring, age-race interaction, UAI in the interval of rectal STI diagnosis/censoring, any reported black partners in the interval of rectal STI diagnosis/censoring, any reported receptive anal intercourse (RAI) for the duration of the study, census-tract-level poverty, diagnosis of any non-rectal STIs for the study duration, and any non-injection drug use for the duration of the study. These covariates, chosen *a priori*, may confound the association of interest as markers of individual risk behaviors or markers of high-prevalence sexual networks, and have been strongly associated with STI and HIV incidence [18,28-30].

The primary confounder of the STI-HIV association, interval-specific UAI, was defined as reporting any UAI partners, condom failure, or inconsistent condom use in the six months prior to rectal STI diagnosis or censoring. For the interval-specific reporting of black partners, missing data in the interval of interest were replaced by data from the most recent interval with data. Non-injection drug use was defined as a positive drug-screen at baseline or self-reported drug use at any interval. Poverty was defined as the 2006–2010 American Community Survey estimate of the percent living in poverty for the census-tract that included the participant’s baseline home address.

Each participant was assigned a weight defined as the stabilized inverse propensity score.

#### Common support assessment

We examined **c**ommon support through visual comparison of the distributions of modeled probabilities stratified by observed rectal STI status [31].

#### Balance assessment

We examined balance of potential confounders by calculating standardized bias. In order to examine the changes in confounder distribution due to IPTW, we calculated standardized biases for the original sample and for the sample following application of IPTW (i.e. the selected method of propensity score application) [31-33].

#### Weighted survival analysis

We then calculated an adjusted HR using IPTW-weighted Cox proportional hazards (PH) regression, modeling incident HIV as a function of the following predictors of HIV incidence and high-prevalence sexual networks: diagnosis with an incident rectal STI, UAI in the interval of HIV diagnosis/censoring (as defined above), any reported black partners in the interval of HIV diagnosis/censoring, and age at HIV diagnosis/censoring [14,18]. The proportional hazards assumption for STI-HIV HR was assessed using visual examination of log-log survival curves and goodness-of-fit testing using Schoenfeld residuals [34]. IPTW-weighted adjusted survival curves were created [35].

All analyses were performed in SAS v9.3 (SAS Institute, Cary, NC) [36]. Code for this analysis is available in Additional file 1. The Institutional Review Board of Emory University approved this study. All participants provided written informed consent prior to enrollment.

## Results

Of 803 men originally enrolled in the study, 562 (70%) had HIV-negative screening results at baseline. Six men were found to be acutely infected with HIV at the three-month visit, leaving 556 men who were truly HIV-negative at baseline and enrolled prospectively. 552 (99%) men had complete data for all covariates and were included in this analysis. Of these men, over the course of the study, 79 (14%) were diagnosed with an incident rectal STI and 26 (5%) men were diagnosed with HIV (Table 1). In 6 men (23%), the incident rectal bacterial STI preceded the HIV infection.

**Table 1:** **Characteristics of individuals with rectal STI and HIV infections among a cohort of Atlanta-area MSM**

	**Incident rectal STI**			**Incident HIV**		
	**Yes (n = 79)**	**No (n = 473)**		**Yes (n = 26)**	**No (n = 526)**	
	**Median (IQR)**	**Median (IQR)**	**OR** ^**1**^ **(95% CI)**	**Median (IQR)**	**Median (IQR)**	**OR** ^**1**^ **(95% CI)**
Age at diagnosis	25.0 (22.1, 28.5)	28.1 (24.4, 33.9)	0.7 (0.5, 0.8)	24.6 (22.5, 28.8)	27.6 (24.3, 33.5)	0.6 (0.4, 1.0)
Poverty	18.2 (9.9, 29.7)	13.8 (8.8, 25.9)	1.1 (1.0, 1.1)	18.8 (10.5, 29.7)	13.9 (8.8, 26.3)	1.1 (0.9, 1.2)
	**N (%)**	**N (%)**	**OR** ^**2**^ **(95% CI)**	**N (%)**	**N (%)**	**OR** ^**2**^ **(95% CI)**
Black race	49 (62)	202 (43)	2.2 (1.3, 3.6)	19 (73)	232 (44)	3.4 (1.4, 8.3)
Ever reporting RAI	67 (85)	326 (69)	2.5 (1.3, 4.8)	21 (81)	372 (71)	1.7 (0.6, 4.7)
Drug use	46 (58)	254 (54)	1.2 (0.7, 1.9)	13 (50)	287 (55)	0.8 (0.4, 1.8)
Black partners^3^	41 (52)	182 (38)	1.7 (1.1, 2.8)	16 (62)	202 (38)	2.6 (1.1, 5.8)
Reported UAI^3^	59 (75)	303 (64)	1.7 (1.0, 2.8)	21 (81)	341 (65)	2.3 (0.8, 6.1)
Non-rectal STI diagnosis^4^	18 (23)	34 (7)	3.8 (2.0, 7.2)	0 (0)	52 (10)	--

^1^Unadjusted OR for a five unit increase in the given variable.

^2^Unadjusted OR for the given variable.

^3^In the interval of diagnosis/censoring.

^4^Urethral GC, urethral CT or syphilis.

In unadjusted analysis, incident HIV was significantly associated with prior incident rectal bacterial STI (HR (95% CI): 3.6 (1.4, 9.2)).

**Figure 2:**
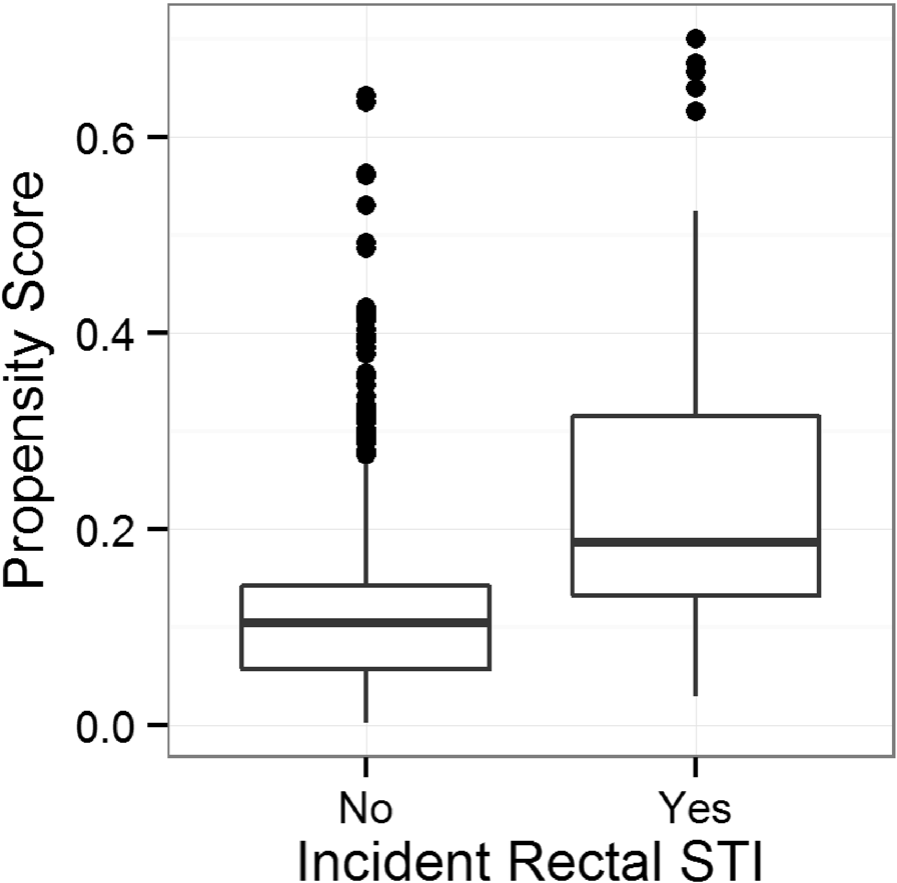
**Evaluation of common support using distributions of propensity scores for each exposure group.**

As expected based on our *a priori* confounding assumptions (Figure 1), the observational nature of the data resulted in different, but overlapping, distributions of propensity scores between exposure groups (Figure 2). This difference indicates the true confounding potential due to the imbalance in these covariates. However, the overlapping ranges indicates that the propensity model exhibits common support.

Application of IPTW resulted in balanced covariates between exposure groups, including those covariates that were strongly unbalanced in the unweighted data (Tables 2 and 3). The standardized biases for all covariates were below the generally accepted 0.25 threshold, and, with the exception of the standardized bias for RAI, were below the more conservative 0.10 threshold, suggesting minimal differences in the weighted distributions between exposure groups following application of IPTW.

**Table 2:** **Distribution of continuous potential confounders by exposure status in the unweighted and weighted study samples**

	**Unweighted**				**Weighted**			
**Variable (Exposure group)**	**Mean (95% CI)**	**Median (IQR)**	**Range**	**Standardized bias**	**Mean (95% CI)**	**Median (IQR)**	**Range**	**Standardized bias**
Age (No STI)	29.6 (29.0, 30.2)	28.1 (24.4, 33.9)	18.2-71.6	−0.45	29.2 (28.6, 29.8)	27.5 (24.0, 32.9)	18.2-71.6	0.04
Age (STI)	26.7 (25.3, 28.1)	25.0 (22.1, 28.5)	19.1-51.4		29.5 (27.9, 31.1)	27.6 (24.5, 33.9)	19.1-51.4	
Poverty (No STI)	18.4 (17.2, 19.6)	13.8 (8.8, 25.9)	0.5-73.9	0.15	18.6 (17.4, 19.9)	13.9 (8.8, 26.3)	0.5-73.9	−0.04
Poverty (STI)	20.4 (17.3, 23.4)	18.2 (9.9, 29.7)	1.5-73.9		18.1 (15.2, 20.9)	14.5 (8.5, 26.6)	1.5-73.9

**Table 3:** **Distribution of categorical potential confounders in the weighted and unweighted study samples**

	**Unweighted**		**Weighted**	
**Variable (Exposure group)**	**Proportion**	**Standardized bias**	**Proportion**	**Standardized bias**
Black race (STI)	62	0.39	47	0.02
Black race (No STI)	43		46	
Reported drug use (STI)	58	0.09	55	<0.01
Reported drug use (No STI)	54		54	
Reported RAI (STI)	85	0.38	79	0.18
Reported RAI (No STI)	69		71	
Reported black partners^1^ (STI)	52	0.27	41	0.02
Reported black partners^1^ (No STI)	38		40	
Reported UAI^1^ (STI)	75	0.23	61	−0.09
Reported UAI^1^ (No STI)	64		66	
Non-rectal STI diagnosis^2^ (STI)	23	0.45	10	0.02
Non-rectal STI diagnosis^2^ (No STI)	7		10	

^1^In the interval of diagnosis/censoring.

^2^Urethral GC, urethral CT or syphilis.

While not all covariates in the propensity model were significantly associated with incident rectal STI (Additional file 2: Table S1), all were retained due to previously observed associations with incident STI and HIV and their association with high-risk sexual networks [18,28-30].

**Figure 3:**
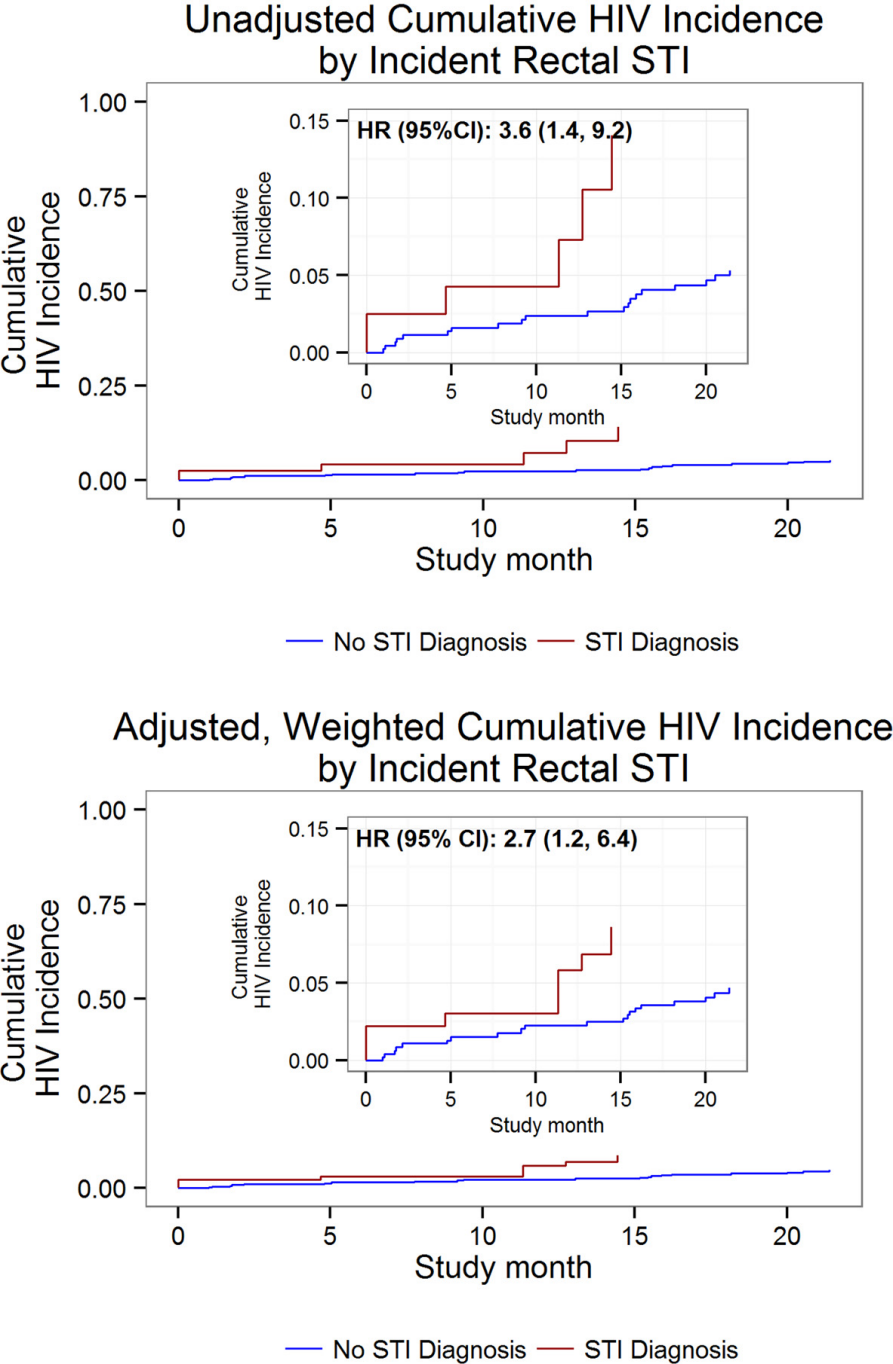
**Unadjusted and adjusted cumulative incidence curves for incident HIV by incident rectal bacterial STI diagnosis.**

In the final weighted and adjusted model, incident rectal STI was significantly associated with incident HIV (HR (95% CI): 2.7 (1.2, 6.4)). Full Cox PH model results are provided in Additional file 2: Table S2. The weighted, adjusted association was both more precise and attenuated (25% reduction) when compared to the crude HR. The attenuation of the HR following control for confounding is evident in the crude and adjusted survival curves (Figure 3).

## Discussion

Our analysis demonstrates the use of propensity score methods to examine the causal effect of rectal bacterial STI infection on HIV seroconversion. This method accounts for time-varying behavioral confounding and maximizes the information obtained from a limited number of events, permitting observational data to simulate an RCT. We found that, in a cohort of initially HIV-negative MSM, controlling for time-varying risk behaviors and time-invariant demographic factors, diagnosis with HIV was significantly associated with prior diagnosis of rectal STI. Among our sample, the time to HIV infection was significantly decreased in men who had been previously diagnosed with rectal STI, compared to those who had not (Figure 3). Our analysis accounts for the primary time-varying confounder of the rectal STI-HIV association, UAI in the interval of STI diagnosis or censoring, and other time-varying and time-invariant confounders. Adjusting for HIV risk factors in the Cox PH model and weighting by the stabilized propensity score attenuated the association and increased its precision as compared to the crude HR. Therefore, our analysis lends support to the association between incident rectal STI and HIV diagnosis being truly causal.

Associations observed in prior studies, which did not include data on specific high-risk behaviors preceding both STI and HIV diagnoses, could have been confounded by deviations from “typical” behavioral patterns [4,6,7]. Prior studies have also relied on self-reported STI diagnoses [5]. Our analysis addressed these limitations, ensuring that temporal relationships between STI infection and its preceding UAI, between HIV infection and its preceding UAI, and between STI infection and HIV infection are accounted for in the analysis and ensuring that both HIV and STI were diagnosed using biological methods.

These potentially changing patterns of behavior and their inclusion as necessary causes of both our exposure and outcome required the use of more complex methods. Additionally, the relatively small number of seroconversions in our cohort limited the number of covariates that could be included in a Cox PH model [37]. The application of propensity model derived IPTW to a Cox PH model solved these analytic challenges by permitting our observational data to approximate a randomized trial, balancing the two exposure groups (in our case, those with and without rectal STI) on measured confounders [8]. Also, propensity models permitted adjustment for a large number of confounders without their direct inclusion in the model. For time-to-event analyses with a small number of events, IPTW maximizes data available while maintaining balance of measured covariates between exposure groups and producing a minimally biased effect estimate [21].

### Limitations

While our analysis addressed many issues with prior analyses, limitations remain. The estimation of causal effect requires no unmeasured confounders and inclusion of all confounders without misclassification [19]. UAI in the interval of rectal STI diagnosis is the primary, overriding confounder of the association in that it is the most proximal, direct behavior by which an individual could acquire both a rectal STI and HIV. We also controlled for other weaker confounders, but cannot rule out the possibility of unmeasured confounders. As we have included the primary source of confounding, we believe that the potential for bias due to unmeasured confounding is low.

There is evidence that UAI is misclassified for some participants in our sample. While UAI was significantly associated with incident STI, it was not significantly associated with incident HIV in this analysis (Additional file 2: Tables S1 and S2). Since UAI is a practically necessary cause of both infections, the odds ratio between UAI and HIV should be infinite, yet such estimates are hardly observed in HIV/STI research, suggesting that UAI misclassification is universal [5,7,38-41]. Based on our data, we believe UAI to be underreported among those infected with STI or HIV, but the direction of misclassification among those who are uninfected is unclear. Additional studies understanding misclassification of this critical HIV risk variable in MSM are needed.

Additionally, given our small number of events, we were unable to explore the role of multiple STIs in HIV acquisition, as others have done [4]. As we selected the first instance of rectal STI, rather than accounting for multiple rectal STI diagnoses, our effect estimates are conservative. Future studies should examine individual STIs and combinations of STIs to further refine this association.

## Conclusions

In support of prior research, our analysis strongly suggests that rectal bacterial STI may be a cause of HIV infection, and not solely a marker of high-risk behaviors. Packaging STI prevention with HIV prevention in MSM may be effective in reducing incidence of both, despite a lack of success of this approach in heterosexuals [42].

In this analysis, we have detailed the use of a propensity score weighted Cox PH model, providing a causal framework for future analyses of HIV incidence. By employing this method, we have approximated the gold standard of study design, the RCT, when such a design is impossible. Given the typically small number of events in HIV incidence studies, propensity score weighting permits adjustment for a large number of time-varying covariates that may be on confounding paths, resulting in a precise effect estimate.

## Additional files

Additional file 1:
**SAS v9.3 code for estimating propensity scores and their application to a Cox PH model.**
Additional file 2: Table S1. Propensity model parameter estimates and estimated OR. **Table S2.** IPTW Cox PH model parameter estimates and estimated HR.

## Abbreviations

HIV: Human immunodeficiency virus
HR: Hazard ratio
IPTW: Inverse probability of treatment weighting
MSM: Men who have sex with men
PH: Proportional hazards
RAI: Receptive anal intercourse
STI: Sexually transmitted infection
UAI: Unprotected anal intercourse
