# Supplementary material for: An application of propensity score weighting to quantify the causal effect of rectal sexually transmitted infections on incident HIV among men who have sex with men
Source: BMC Med Res Methodol. 2015 Mar 21;15:25. doi: 10.1186/s12874-015-0017-y (PMC4369368; doi:10.1186/s12874-015-0017-y)
Supplement: Additional file 1: — SAS v9.3 code for estimating propensity scores and their application to a Cox PH model. [file 12874_2015_17_MOESM1_ESM.pdf]

Supplemental File: SAS v9.3 code for estimating propensity scores and their application to a Cox PH model

In the SAS code below, *ds* represents a dataset containing the exposure (*sti*), outcome (*hiv*), person-time (*hiv_pt*), and covariates. For this code, variables *var1* (continuous) and *var2* (dichotomous) are potential confounders occurring prior to exposure. Variables *var3* and *var4* are covariates that occur prior to the outcome. This code assumes that all dichotomous variables are coded as 1 for the presence of the variable and 0 for its absence.

```
/* First model the exposure of interest (i.e. the treatment) as a function of
potential confounders. */
/* The output dataset ps_p contains the IPTW weights */
proc logistic data = ds;
      model sti (ref = '0') = var1 var2/
            lackfit outroc = ps_r1a;
            output out= ps_p XBETA=beta STDXBETA= betasd PREDICTED = ps_pred;
run;

/* Calculate weights based on predicted probabilities*/
data ps_weight;
      set ps_p;
      if sti = 1 then wt_ps_pred=ps_pred;
      else wt_ps_pred = 1-ps_pred;
run;

/* Calculate a mean weight by exposure group in order to standardize weights
*/
proc sort data=ps_weight;
      by sti;
proc means data = ps_weight noprint;
      var wt_ps_pred;
      by sti;
      output out = q mean = mn_wt;
run;

/* Stabilize the weights with the mean for each treatment group*/
/* The variable wt2 is the standardized IPTW weight */
data ps_weight2;
      merge q ps_weight;
      by sti;
      wt2 = mn_wt/wt_ps_pred;
      drop _type_ _freq_;
run;

/* Propensity Score Diagnostics*/
/* Evaluate common support by comparing the distributions of propensity
scores */
proc sort data = ps_weight2;
      by sti;
proc boxplot data=ps_weight2;
      symbol width = 2;
      plot ps_pred*sti/
      cboxes=black
      cframe = white
      idsymbol = circle
      idcolor = black
```

```
      font='times new roman' height=3.5
      boxwidth=6
      boxstyle=schematic
      waxis = 2;
run;

/* Evaluate balance by comparing weighted distributions of
individual continuous variables */
proc means data = ps_weight2 min lclm q1 mean median q3 uclm max
      maxdec=1 fw=6 nmiss;
      class sti;
      var var1;
      weight wt2;
run;


/* Evaluate balance by comparing weighted distributions of individual
dichotomous variables */
proc freq data = ps_weight2;
      tables var2*sti;
      weight wt2;
run;

/* After verifying the PH assumption, run the Cox PH model */
/* Unadjusted Cox PH model */
title "Unadjusted Cox Model";
proc phreg data=ps_weight2;
      class sti (ref="0" param=ref) /descending;
      model hiv_pt*hiv(0)= sti/rl;
run;

/* Adjusted, weighted Cox PH Model */
title "Adjusted Cox Model";
proc phreg data=ps_weight2;
      class sti /descending;
      model hiv_pt*hiv(0)= sti var3 var4 /rl;
      weight wt2;
      estimate "HR" sti 1/exp;
run;
```
