# Supplementary material for: An application of propensity score weighting to quantify the causal effect of rectal sexually transmitted infections on incident HIV among men who have sex with men
Source: BMC Med Res Methodol. 2015 Mar 21;15:25. doi: 10.1186/s12874-015-0017-y (PMC4369368; doi:10.1186/s12874-015-0017-y)
Supplement: Additional file 2: Table S1. — Propensity model parameter estimates and estimated OR. Table S2. IPTW Cox PH model parameter estimates and estimated HR. [file 12874_2015_17_MOESM2_ESM.pdf]

Supplemental Table A. Propensity model parameter estimates and estimated OR

Variable	Parameter estimate (SE)	Adjusted OR (95% CI)[1]
Black race	4.52 (1.40)	--
Age at STI diagnosis	0.003 (0.026)	--
Race-age interaction	-0.14 (0.05)	--
White, one year increase in age	--	1.0 (1.0, 1.1)
Black, one year increase in age	--	0.9 (0.8, 1.0)
Poverty	0.004 (0.010)	1.0 (1.0, 1.0)
Reported drug use	0.29 (0.27)	1.3 (0.8, 2.3)
Reported RAI	0.95 (0.35)	2.6 (1.3, 5.2)
Reported black partners[2]	-0.05 (0.35)	0.9 (0.5, 1.9)
Reported UAI[2]	0.65 (0.30)	1.9 (1.1, 3.4)
Non-rectal STI diagnosis[3]	1.18 (0.36)	3.2 (1.6, 6.5)

[1] Adjusted OR for a once unit increase in the listed variable.
[2] In the interval of STI diagnosis/censoring
[3] Urethral GC, urethral CT or syphilis

Supplemental Table B. IPTW Cox PH model parameter estimates and estimated HR

Variable	Parameter estimate (SE)	Adjusted HR (95% CI)[1]
Incident rectal STI	1.00 (0.43)	2.7 (1.2, 6.4)
Age at HIV diagnosis	-0.10 (0.04)	0.9 (0.8, 1.0)
Reported black partners[2]	0.69 (0.38)	2.0 (1.0, 4.1)
Reported UAI[2]	0.45 (0.43)	1.6 (0.7, 3.7)

[1] Adjusted, weighted HR for a once unit increase in the listed variable.
[2] In the interval of HIV diagnosis/censoring
